# The joint role of the immune microenvironment and N^7^-methylguanosine for prognosis prediction and targeted therapy in acute myeloid leukemia

**DOI:** 10.3389/fgene.2025.1540992

**Published:** 2025-06-13

**Authors:** Zhixiang Chen, Zhimei Chen, Xiaobo Huang, Xiongbin Yan, Xiaolin Lai, Shaoyuan Wang

**Affiliations:** ^1^ Department of Hematology, Fujian Institute of Hematology, Fujian Provincial Key Laboratory on Hematology, Fujian Medical University Union Hospital, Fuzhou, China; ^2^ Union Clinical Medical Colleges, Fujian Medical University, Fuzhou, China; ^3^ Department of Radiology, Xianyou County General Hospital, Putian, China; ^4^ Department of Gastric Surgery, Fujian Medical University Union Hospital, Fuzhou, China; ^5^ Key Laboratory of Ministry of Education of Gastrointestinal Cancer, Fujian Medical University, Fuzhou, China; ^6^ Fujian Key Laboratory of Tumor Microbiology, Fujian Medical University, Fuzhou, China

**Keywords:** acute myeloid leukemia, immune microenvironment, m7G, gene signature, prognosis, therapy

## Abstract

**Background:**

The tumor immune microenvironment (TIME) and N^7^-methylguanosine (m7G) modification play crucial roles in the progression of acute myeloid leukemia (AML). This study aims to establish an IME-related and m7G-related prognostic model for improved risk stratification and personalized treatment in AML.

**Methods:**

Immune score for the Cancer Genome Atlas acute myeloid leukemia (AML) patients were calculated using the ESTIMATE algorithm, followed by identification of immune score-associated differentially expressed genes Non-negative matrix factorization (NMF) clustering was performed to stratify AML subtypes based on immune microenvironment (immune microenvironment)-related DEGs and 29 m7G regulatory genes. Intersecting DEGs co-linked to IME and m7G features were analyzed through weighted gene co-expression network analysis Weighted correlation network analysis combined with univariate Cox, LASSO, and multivariate Cox regression to establish a prognostic signature. Biological pathway disparities between risk subgroups were analyzed via Gene Set Enrichment Analysis, Gene Set Variation Analysis, and ssGSEA. A clinical nomogram integrating the signature with prognostic indicators was developed. The expression of the 12 prognostic genes were tested and compared in AML and healthy donors. Drug sensitivity predictions for high-risk patients were generated using oncoPredict, supported by molecular docking simulations of ligand-target interactions and *in vitro* validation of candidate compounds in AML cell models.

**Results:**

We constructed an IMEm7G prognostic signature comprising 12 genes (MPZL3, TREML2, PTP4A3, AHCYL1, CBR1, REEP5, PPM1H, WDFY3, LAMC3, KCTD1, DDIT4, KBTBD8) that robustly stratified AML risk and predicted survival in multiple cohorts. The high- and low-risk subgroups exhibited divergent biological pathways, mutational landscapes, immune infiltration patterns, immune checkpoint expression, and HLA profiles. This signature further guided therapeutic selection, with dactolisib identified as a high-risk-specific candidate. The quantitative real-time PCR (qPCR) analysis demonstrated that in comparison with healthy donors, the expression of WDFY3, PPM1H, and REEP5 was significantly lower, while that of PTP4A3, AHCYL1, CBR1, MPZL3, TREML2, and KBTBD8 was higher in AML patients. *In vitro* CCK-8 assays validated dactolisib’s monotherapy efficacy and synergistic cytotoxicity when combined with doxorubicin in AML cells.

**Conclusion:**

The IMEm7G gene signature established in our study effectively optimized the risk classification and predicted immunotherapy response in AML. Moreover, dactolisib was identified and demonstrated cytostatic activity alone and synergistic effects with doxorubicin in AML cells.

## Introduction

Acute myeloid leukemia (AML) is a hematologic malignancy with high mortality that is characterized by impaired hematopoiesis and extramedullary infiltration of immature myeloid hematopoietic cell invasion. It is one of the most common types of leukemia in adults (Tallman et al., 2019). Induction chemotherapy combined with consolidation chemotherapy or hematopoietic stem cell transplantation (HSCT) has been the primary treatment strategy for most *de novo* AML patients over recent decades (Rai et al., 1981; Short et al., 2020). Although many patients experience their first remission after the standard treatment, the 5-year survival rate remains only 30% for young patients and less than 10% for older patients (Miller et al., 2019). The immune microenvironment (IME) of AML significantly impacts therapeutic efficacy and prognosis (Tettamanti et al., 2022). Additionally, the intricate outcomes of AML patients vary with alterations in epigenetic factors (Ueda and Steidl, 2021; Zhao et al., 2024; Zhang et al., 2024). Identifying meaningful biomarkers based upon the IME and its epigenetic characteristics to stratify patients is a wise strategy for exploring heterogeneity, providing prognostic information, and guiding therapy.

The tumor immune microenvironment (TIME) is a complex regulatory network comprised of tumor cells and surroundings, and the surroundings mainly include immune cells and stromal cells. The immune landscape within the tumor microenvironment has brought new insights into treatment approaches for AML patients and has also facilitated the development of immunotherapy (Vago and Gojo, 2020). Nonetheless, accurately identifying the optimal population that would benefit from immunotherapy remains a significant concern, requiring more comprehensive risk stratification and a deeper understanding of the IME for patients with AML.

Epigenetics involves studying how gene expression is regulated without changes to nucleotide sequence. Researches has indicated that dysregulated RNA modifications frequently result in aberrant gene expression and are closely correlate with developmental disorders and malignancies (Cavalli and Heard, 2019). N7-methylguanosine (m7G) is one of the most common RNA modifications that arises from the methylation of RNA guanine at the N7 position by methyltransferase and is commonly found in transfer RNAs (tRNAs), ribosomal RNAs (rRNAs), the 5′cap of messenger RNA (mRNA), and within mRNAs themselves (Cai et al., 2023). Recent studies have demonstrated that the m7G modification has the potential to influence the IME, and this alteration can subsequently contribute to tumor development and therapeutic effectiveness (Han et al., 2024). Nonetheless, no research has been done about integrating both IME and m7G for prognostic stratification and treatment guidelines in AML. Our study aimed to explore the feasibility of concurrently modeling these two critical components to enhance our understanding and improve the management of AML.

In this study, we constructed a 12-gene signature based upon both IME and m7G for robust risk stratification. Moreover, we established a nomogram incorporating common clinical indices and the gene signature. We ultimately confirmed that dactolisib has therapeutic potential, demonstrating inhibitory activity against AML cells in monotherapy and exhibiting a synergistic effect when combined with doxorubicin.

## Materials and methods

### Data acquisition and processing

AML patients with complete clinical characteristics were enrolled in our study. The transcriptomic profiles of 151 AML patients were retrieved from the TCGA cohort. The clinical data of AML patients was gathered from the UCSC Xena database (http://xena.ucsc.edu/). The transcriptomic profiles of Beat-AML (346 AML samples) and the microarray data with matrix format of AML patients from GSE10358 (91 AML samples) and GSE71014 (104 AML samples) were used for external validation. The probes for the GSE10358 and GSE71014 were annotated by Affymetrix Human Genome U133 Plus 2.0 Array GPL platform and Illumina HumanHT-12 V4.0 expression beadchip GPL10558 platform, respectively. If multiple probes corresponded to the same gene, the mean expression value was calculated and defined as a final value using the limma (Ritchie et al., 2015) package in the R language (version 4.4.1). The transcriptome data from TCGA was analyzed in FPKM values.

### Identification of DEGs related to AML immune microenvironment (IME)

The immune score, stromal score, and estimate score of 151 AML cases from the TCGA database were assessed using the ESTIMATE algorithm (Yoshihara et al., 2013). Patients were classified into high and low immune score groups based on the median immune score. We used the limma package to compare the DEGs between the high- and low-score groups, with an adjusted p-value <0.05 and | log2 fold change (FC) | > 1 as the cutoff criteria. The Gene Ontology (GO) and Kyoto Encyclopedia of Genes and Genomes (KEGG) pathway enrichment analyses of DEGs were conducted with the “clusterProfiler” R package (Yu et al., 2012). The “ggplot2” was utilized to visualize the outcome (Ginestet, 2011).

### Identification of IME-relevant and m7G-relevant subclasses

For IME-relevant unsupervised classification, the IME-related DEGs were enrolled for non-negative matrix factorization (NMF) clustering (Gao and Church, 2005). 29 m7G-related genes were identified from the published literature (Tomikawa, 2018) and the Gene Set Enrichment Analysis (GSEA) website (http://www.gsea-msigdb.org/gsea/index.jsp). AML patients from the TCGA database were divided into subgroups based on the expression of 29 m7G-related genes using the “NMF” R package. The “survival” R package was used for the Kaplan-Meier (KM) complete survival curve analysis between the different clusters.

### Weighted correlation network analysis

Using the WGCNA package in R, the Weighted correlation network analysis (WGCNA) determines the correlation patterns between genes (Langfelder and Horvath, 2008). We executed WGCNA using 2429 IME- and m7G-relevant DEGs (named IMEm7G-DEGs) to identify the ‘survival’ models for further investigation. The correlation values of DEGs to ‘survival’ models were determined through Pearson’s correlation coefficients. The soft thresholding power was determined by evaluating both scale independence (*R* ^2^) and mean connectivity. A threshold was selected to ensure a scale independence (*R* ^2^) value greater than 0.8 while maintaining a sufficiently high mean connectivity, thereby preserving biologically relevant information. Based on these criteria, β = 4 was identified as the optimal value for our analysis. The adjacency matrix was transformed into a topological overlap matrix (TOM) for gene hierarchical cluster analysis to generate co-expression modules. Similarly expressed genes were grouped into the same gene module. The minimum module size was defined as 30 genes, and modules with a dissimilarity of <0.3 were merged. We finally mined the gene models related to survival time and survival status.

### Construction of an IMEm7G-related gene signature

The survival-correlated gene models (including survival time correlated or survival status correlated; p < 0.05) were obtained from WGCNA analysis, and genes in these models were screened for univariate Cox regression analysis. Meanwhile, a Kaplan-Meier (KM) survival analysis was conducted among these genes to explore the effect of each gene on AML survival.

To optimize the Cox model, we performed the least absolute shrinkage and selection operator (LASSO) regression analysis by the glmnet (Friedman et al., 2010) package in R. The selected genes were later analyzed using multivariate Cox regression to establish an IMEm7G-based gene signature. In the gene signature, an individual’s risk score was calculated using the formulas: risk score = β1 * Gene1 expression + β2 * Gene2 expression + . + βn * Genen expression, in which β represents the regression coefficient of each variable.

The median risk score was used to categorize patients into high-/low-risk groups. The reliability of the gene signature for predicting the 1-, 2-, 3-, 4-, and 5-year survival was evaluated via the time-dependent receiver operating characteristic (ROC) curve by survivalROC (https://cran.r-project.org/package=survivalROC) package under R environment. The survival (https://cran.r-project.org/package=survival) and survminer (https://cran.r-project.org/package=survminer) packages were implemented to perform the KM survival analysis and Cox models. A p-value <0.05 was a significant statistical difference in survival prognosis.

### Verification of the robustness of the 12-gene signature

Three additional cohorts of AML patients from Beat-AML, GSE10358, and GSE71014 were used as validation cohorts. Based on the gene signature, we calculated the risk score, overall survival (OS) rates, and area under the curves (AUCs) values of the distinct AML cohorts separately.

### Functional analyses of the low- and high-risk groups

The GSEA was performed on high- and low-risk groups to identify enriched principal pathways (GSEA 4.1.0) (Subramanian et al., 2005). The javaGSEA Desktop Application was downloaded from https://www.gsea-msigdb.org/gsea/index.jsp. We used the Hallmark sets (Liberzon et al., 2015) as reference sets. The analysis was carried out via 1,000 permutations. The pathways with a |normalized enrichment score (NES)| > 1, p-value (NOM p-value) < 5%, and q-value (FDR q-value) < 25% were statistically significant. The enrichment pathways for variations between subtypes were evaluated through Gene Set Variation Analysis (GSVA) (Hänzelmann et al., 2013).

### Deciphering the immune landscape of AML patients via the ssGSEA algorithm

The single sample GSEA (ssGSEA) was adopted with the GSVA package to depict the immune infiltration characteristics of distinct immune cell infiltrations in AML samples.

### Establishment of a nomogram with clinical application

With the replot (https://CRAN.R-project.org/package=regplot) package, the 12-gene signature was combined with clinical features, including age, cytogenetics, and transplantation treatment, to establish a nomogram. We ran univariate and multivariate Cox regression analyses for the Hazard ratios (HR) of distinct factors in the nomogram. The AUC values, decision curve analysis (DCA), concordance index (C-index), and calibration curves were applied to evaluate the practicality of the nomogram.

### Drug prediction analysis

The “oncoPredict” R package was employed to identify the therapeutic medicines for AML patients based on the genomics of drug sensitivity in cancer (GDSC) database (https://www.cancerrxgene.org/) (Maeser et al., 2021). We performed molecular docking of the potential therapeutic ingredients and the predicted targets with unfavorable events, including PTP4A3, AHCYL1, TREML2, CBR1, and MPZL3. The protein structures for these specific genes were retrieved from the PDB database (http://www.rcsb.org/). The PubChem database was utilized to obtain the chemical structures of distinct medicines. The AutoDock tool was utilized to verify the charge balance and identify the rotatable bonds of small molecules (Morris et al., 2009). We performed molecular docking between the targeted proteins and medications using AutoDock Vina (Trott and Olson, 2010). The binding energy obtained from molecular docking experiments served as a docking score to evaluate the protein-ligand binding potential. Combinations of targets and ligands with a selection value of ≤ -5 were deemed to exhibit moderate to strong binding potential. A lower binding energy indicates a stronger interaction between the molecules. The docking results were visualized using PyMol 2.4 software (Bramucci et al., 2012).

### Patient samples

Bone marrow mononuclear cells were obtained from 50 newly diagnosed AML patients at Fujian Medical University Union Hospital (FMUUH), while peripheral blood samples were collected from 20 healthy donors. This study was approved by the FMUUH Ethics Committee and conducted in accordance with the Declaration of Helsinki.

### RNA isolation and quantitative real-time PCR

Total RNA was isolated using TRIzol reagent (TransGen Biotech, China) following the manufacturer’s instructions. Subsequently, 1 μg of total RNA was reverse-transcribed into complementary DNA (cDNA) using a commercial reverse transcription kit (Thermo Fisher Scientific, United States). Quantitative real-time PCR (qPCR) analysis was conducted using SYBR Green PCR Master Mix (Vazyme, China) on a real-time PCR detection system. The relative gene expression levels were determined using the comparative 2^−ΔΔCT^ method (Livak and Schmittgen, 2001). All primer sequences used in this study are provided in Supplementary Table S1.

### Cell counting kit-8 (CCK-8) assay and treatment combination analysis

The cell viability was assessed by CCK-8 assay (APExBIO, United States). The AML cells, including THP-1 (icellbioscience, cat#iCell-h213, China), HL-60 ((icellbioscience, cat#iCell-h098, China), and MOLM-13 (icellbioscience, cat#iCell-h423, China) were seeded in 96-well plates with 100 μL volume per well, following treatment with dactolisib or the combination of dactolisib and doxorubicin for 48 h. For monotherapy, the THP-1 cells were treated with increasing concentrations of dactolisib at 0, 0.1, 0.25, 0.5, and 1 µM. HL-60 cells were treated with dactolisib at 0, 0.1, 0.25, 0.5, 1, and 2 µM concentrations. MOLM-13 cells were treated with dactolisib at 0, 0.05, 0.1, 0.25, and 0.5 µM concentrations.

For the combination treatment, AML cells were treated with the increased concentrations of dactolisib, along with various concentrations of doxorubicin: THP-1 cells received 0, 0.05, 0.1, and 0.2 µM; HL-60 cells received 0, 0.01, 0.02, 0.05, 0.1, and 0.2 µM; and MOLM-13 cells received 0, 0.005, 0.01, 0.02, and 0.05 µM. After 48 h, the cells were incubated with 10 µL of CCK-8 reagent per well for 2 h at 37°C, away from light. The optical density (OD) of each well at 450 nm was measured using a microplate reader (Bio Tek, United States). Cell viability was calculated using the formula: viability (%) = [(treated - blank)/(control - blank)] × 100%. The combination effects and synergy scores of dactolisib and doxorubicin were analyzed with the Synergyfinder, utilizing the Bliss independence model (Ianevski et al., 2020).

## Results

### Clustering of AML patients based on immune microenvironment (IME)- and m7G-relevant genes

**FIGURE 1:**
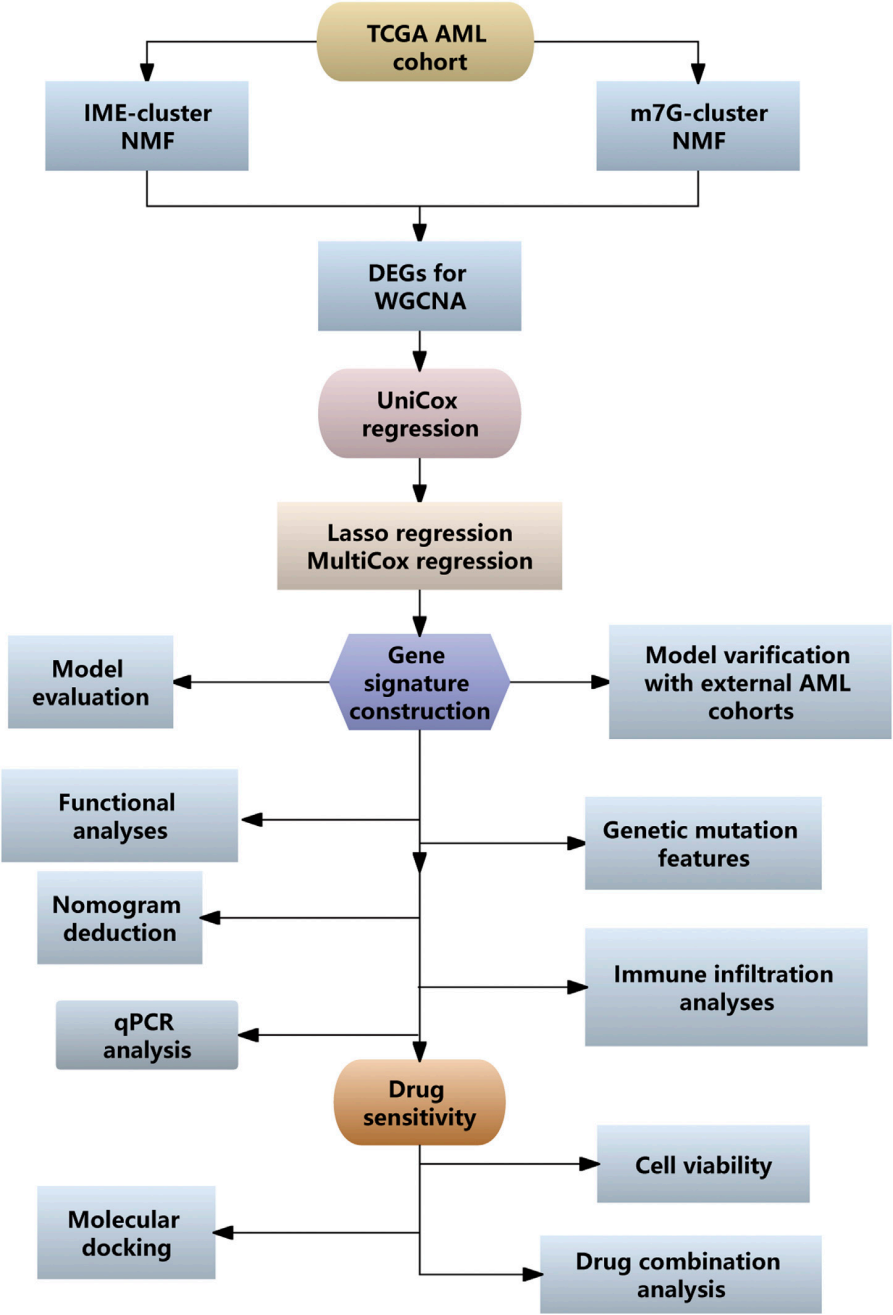
Workflow diagram illustrating the analysis process for the development of a prognostic model associated with IME and m7G.

**FIGURE 2:**
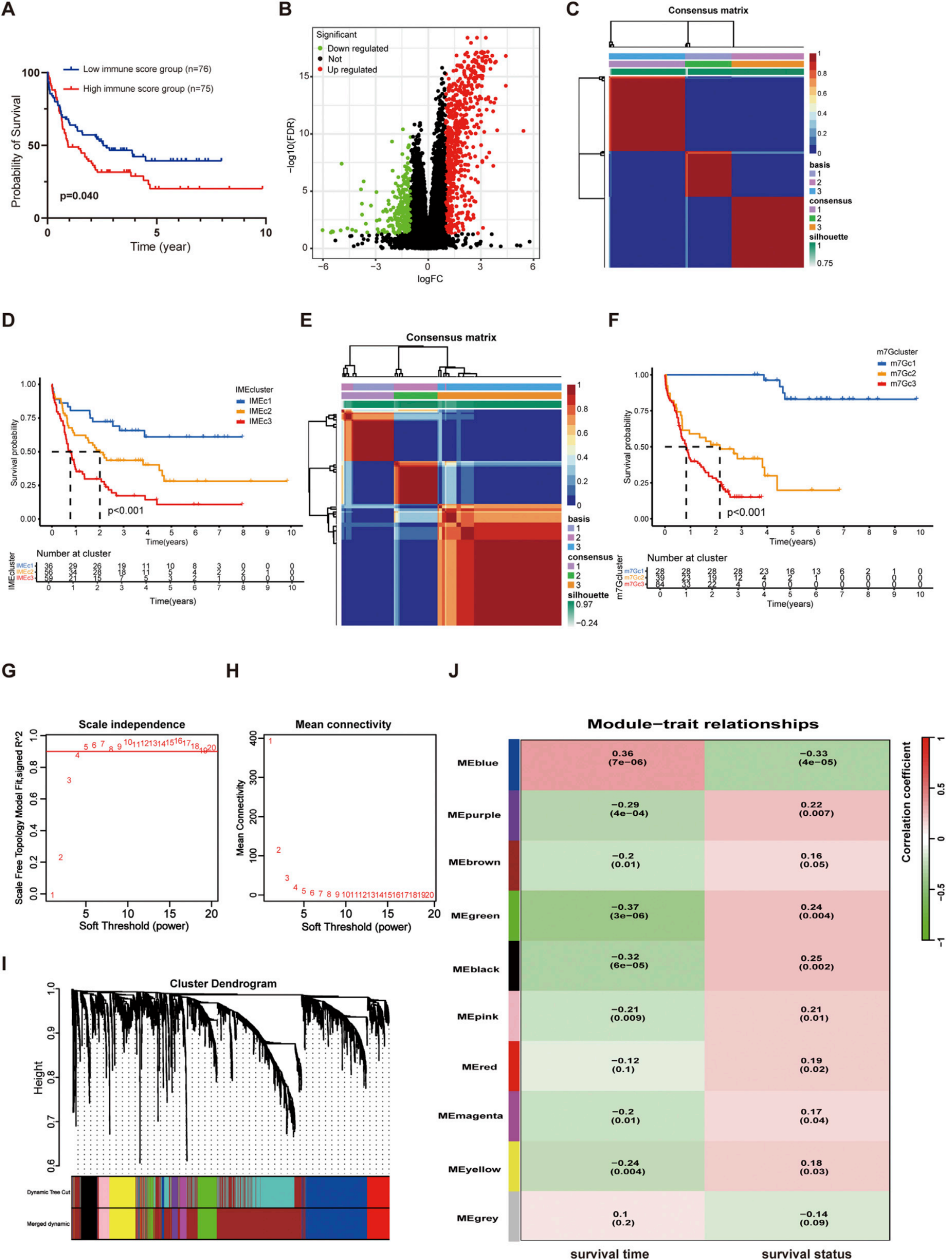
Classification of AML patients via NMF analysis. **(A)** KM curves for OS in the high and low immune score groups. **(B)** Volcano map of DEGs in the high and low immune score groups. **(C)** Heatmap of sample clustering when k = 3 based on the IME NMF analysis. **(D)** KM curves of Cluster1, Cluster2, and Cluster3 in the TCGA AML dataset based on the IME NMF analysis. **(E)** Heatmap of sample clustering when k = 3 based on the m7G NMF analysis. **(F)** KM curves of Cluster1, Cluster2, and Cluster3 in the TCGA AML dataset based on the m7G NMF analysis. **(G)** Analysis of the scale-free fit index for various soft-thresholding powers (β). **(H)** Analysis of the mean connectivity for various soft-thresholding powers. **(I)** The cluster dendrogram of genes in TCGA is depicted here. Each branch in the figure represented a single gene, while each color below corresponded to a co-expression module. **(J)** Heatmap of the correlation between module eigengenes and the survival (including survival time and survival status) of AML.

The procedure of our study was depicted in Figure 1. The immune score, estimate score, and stromal score of each AML patient were calculated using the ESTIMATE algorithm. The Kaplan-Meier (KM) survival analysis indicated that patients with low immune score had better OS than those with high immune score (Figure 2A). We then divided the AML cases into two groups according to the median immune score to investigate the IME-relevant DEGs. In contrast with the low-immune score group, we identified 645 upregulated genes and 273 downregulated genes in the high-immune score group (Figure 2B). The GO and KEGG analyses revealed that these DEGs were primarily enriched in immune and inflammatory processes (Supplementary Figure S2A, B). We subsequently used these DEGs to construct a risk model employing uniCox, Lasso, and multiCox regression analyses. The findings suggested that the model performed well with AUCs over 0.85 (Supplementary Figure S2C). However, we regretfully found that the predictive model showed weak performance in the external AML cohorts (Supplementary Figure S2D–F). Correspondingly, we constructed the m7G-relevant model using 29 m7G-related genes, while the AUCs for the model were less than 0.8 (Supplementary Figure S2G). Meanwhile, the predictive model also revealed poor performance in the external AML cohorts (Supplementary Figure S2H–J). We propose this discrepancy could stem from cross-cohort variability and the exclusive focus on unimodal biomarkers (e.g., microenvironment or RNA modifications), potentially insufficient to deconvolute the disease’s multifactorial nature while limiting model generalizability.

The correlation between m7G and immunity has been reported previously, while few studies have combined the two to optimize the prognostic stratification in AML. We attempted to combine the IME and m7G for modeling and performance evaluation. The IME-related DEGs were used for the AML cluster with NMF analysis. The results showed that AML patients in the TCGA cohort were clustered into three subtypes (named IMEc1, IMEc2, and IMEc3 respectively) (Figure 2C; Supplementary Figure S2K). Patients in IMEc1 demonstrated superior OS rates compared to IMEc2 and IMEc3 (Figure 2D). Meanwhile, we divided patients into three clusters based on the 29 m7G-related genes (named m7Gc1, m7Gc2, and m7Gc3 respectively) (Figure 2E; Supplementary Figure S2L). Patients in m7Gc2 and m7Gc3 exhibited worse OS compared to m7Gc1 (Figure 2F). Interestingly, we found that patients in the m7Gc2 and m7Gc3 showed similar survival probabilities over 1–3 years compared to patients in the IMEc2 and IMEc3. Additionally, we discovered that the m7Gc2 and m7Gc3 exhibited higher immune score when compared to m7Gc1 (Supplementary Figure S2M). This suggested that patients in m7Gc2 and m7Gc3 were characterized by more immune infiltrating cells to impact the OS. We compared the sample information between IMEc1 and m7Gc1 and identified 12 patients who consistently exhibited a favorable prognosis when classified by both methodologies (designated as the FF group). Similarly, 99 patients were consistently classified as having a poorer prognosis by both approaches (designated as the WW group). The comprehensive baseline characteristics of both cohorts are summarized in Supplementary Table S2.

Then, the DEGs between the FF groups and WW groups were identified with an adjusted p-value <0.05 and | log2 FC | > 0.5 as the cutoff criteria, resulting in the screening of 2,429 DEGs for inclusion in WGCNA analysis. Based on the gene expression profiles of individual patients, we defined a threshold value of 10,000, which led to the identification and subsequent exclusion of two outlier samples (Supplementary Figure S2N). A scale-free co-expression network was constructed with a soft threshold power β = 4 (Figures 2G,H). These DEGs were categorized into ten models along with survival time and status (Figures 2I,J). Most gene models were correlated with survival time or survival status with a screening threshold of p < 0.05. In these models, 623 genes were predictive about survival (survival time or survival status) with a p-value <0.05 (Supplementary Table S3).

### Development of the IME and m7G (IMEm7G) related signature for prognostic classification of AML patients

**FIGURE 3:**
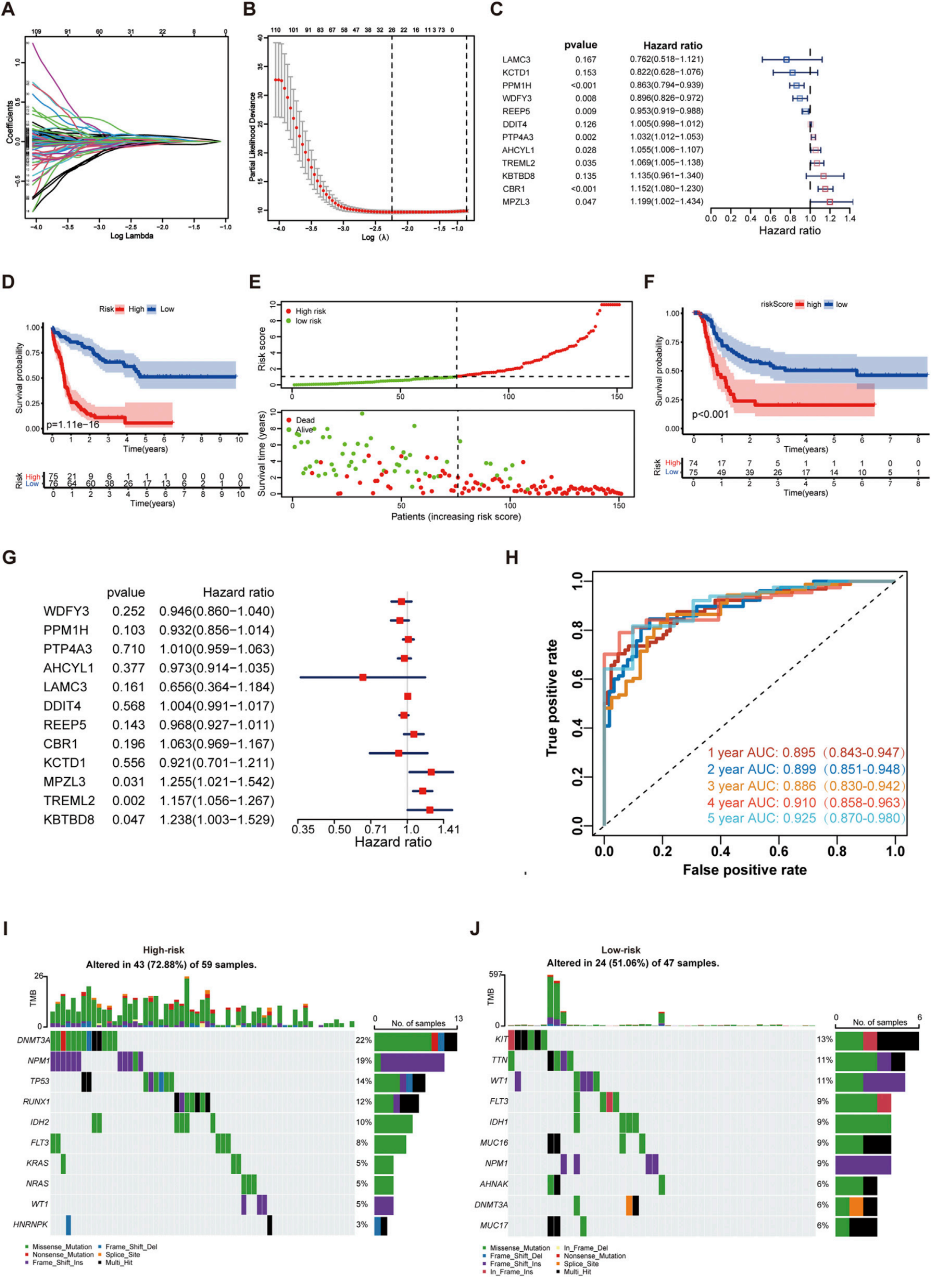
Development of the IME and m7G (IMEm7G) related signature in AML. **(A)** Lambda trajectory of DEGs. **(B)** Confidence interval under lambda. **(C)** Twelve genes were selected for constructing a gene signature using the multivariate Cox regression model. **(D)** KM survival curves of the model predicting the prognostic rate of OS between low- and high-risk groups. **(E)** Scatter plot of the model in the TCGA AML set dividing the samples into high-risk and low-risk groups. **(F)** KM survival curves of the model predicting the prognostic rate of EFS between low- and high-risk groups. **(G)** The expression of TREML2, MPZL3, and KBTBD8 was considered the independent EFS index. **(H)** ROC curves of the risk model for predicting 1-, 2-, 3-, 4-, and 5-year survival in the TCGA AML cohort. **(I)** The top ten gene mutations in the high-risk group. **(J)** The top ten gene mutations in the low-risk group.

The prognosis-related 623 DEGs screened from WGCNA were subjected to univariate Cox proportional hazard regression analysis. The result showed that 579 of 623 genes were highly significant with p-value < 0.05 (Supplementary Table S4). These genes were subsequently incorporated into the LASSO regression analysis to avoid overfitting, and 26 genes were obtained (Figures 3A,B). Using multivariate Cox regression analysis for the 26 genes, 12 prognostic genes for the gene signature were identified. In these genes, MPZL3, TREML2, PTP4A3, AHCYL1, and CBR1 were positively related to poor prognosis (HR > 1 and p < 0.05), whereas REEP5, PPM1H, and WDFY3 were negatively correlated (HR < 1 and p < 0.05) (Figure 3C). Multicollinearity was evaluated using variance inflation factor (VIF) (threshold ≥2 indicating multicollinearity), and all genes showed VIF <2 (Supplementary Table S5). The PH assumption was satisfied for all variables in the Cox model, as confirmed by the global Schoenfeld test (Supplementary Table S6). We subsequently calculated a risk score for each patient using these genes’ expression and multivariate Cox regression coefficients. The risk score was determined by the following formula: Risk score = (−0.2722 * LAMC3 expression) + (−0.1964 * KCTD1 expression) + (−0.1468 * PPM1H expression) + (−0.1097* WDFY3 expression) + (−0.0485 * REEP5 expression) + 0.0054 * DDIT4 expression +0.0319 * PTP4A3 expression +0.0539 * AHCYL1 expression +0.0670 * TREML2 expression +0.1265* KBTBD8 expression +0.1418 * CBR1 expression +0.1814 * MPZL3 expression. The KM analysis indicated that patients in the high-risk group showed poorer clinical outcomes than those in the low-risk group (Figure 3D). As the risk score increased, the survival time for patients decreased (Figure 3E). Additionally, patients with high-risk score showed worse event-free survival (EFS) as well (2 patients who lacked the EFS information were excluded) (Figure 3F). With the multiCox analysis, the expression of TREML2, MPZL3, and KBTBD8 was considered the independent EFS index (Figure 3G).

The AUCs of the risk signature for predicting 1-, 2-, 3-, 4-, and 5-year survival were 0.895, 0.899, 0.886, 0.910, and 0.925, respectively (Figure 3H). Furthermore, we found that patients in the high-risk group were characterized as elderly cohorts with intermediate or poor cytogenetics and less transplantation treatment (Supplementary Table S7). The molecular abnormalities were prevalent in AML, and numerous genetic mutations were associated with poorer prognosis for patients. For profiling the mutation landscape in high- and low-risk groups, we employed the “maftools” and discovered that individuals in the high-risk group exhibited more gene mutations (Figures 3I,J). The top ten mutated genes in the high-risk group were DNMT3A, NPM1, TP53, RUNX1, IDH2, FLT3, KRAS, NRAS, WT1, and HNRNPK. The top ten mutated genes in the low-risk group were KIT, TTN, WT1, FLT3, IDH1, MUC16, NPM1, AHNAK, DNMT3A, and MUC17.

### The performance of the 12-gene signature in the external AML cohorts

**FIGURE 4:**
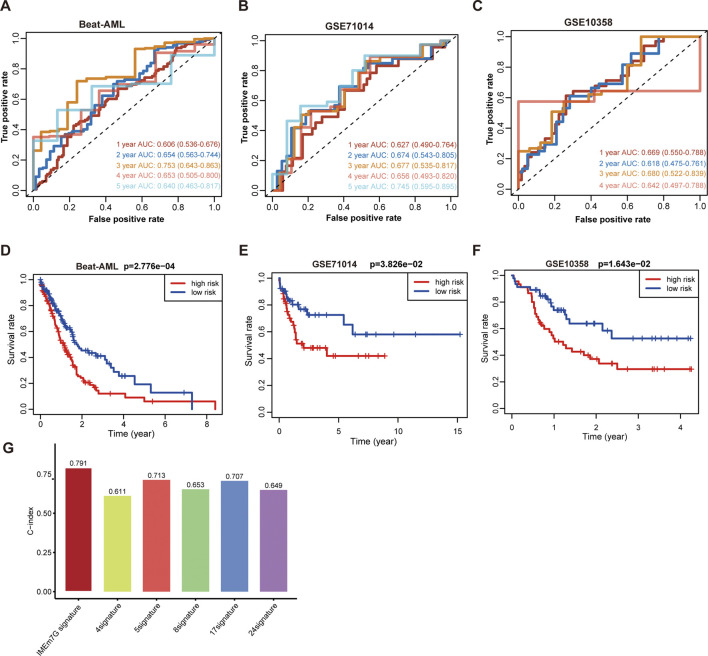
The performance of the 12-gene signature in the external AML cohorts. **(A)** ROC curves of the risk model for predicting 1-, 2-, 3-, 4-, and 5-year OS in the Beat-AML cohort. **(B)** ROC curves of the risk model for predicting 1-, 2-, 3-, 4-, and 5-year OS in the GSE71014 AML cohort. **(C)** ROC curves of the risk model for predicting 1-, 2-, 3-, and 4-year OS in the GSE10358 AML cohort. **(D)** KM survival curves of the model predicting the prognostic rate of OS between low- and high-risk groups in the Beat-AML cohort. **(E)** KM survival curves of the model predicting the prognostic rate of OS between low- and high-risk groups in the GSE71014 AML cohort. **(F)** KM survival curves of the model predicting the prognostic rate of OS between low- and high-risk groups in the GSE10358 AML cohort. **(G)** The C-index of the distinct models for the TCGA AML cohort.

The Beat-AML cohorts, GSE71014 and GSE10358 were used as testing groups. The AUCs in the Beat-AML for predicting 1-, 2, 3-, 4-, and 5-year survival was 0.606, 0.654, 0.753, 0.653, and 0.640, respectively (Figure 4A). The AUCs in the GSE71014 for predicting 1-, 2, 3-, 4-, and 5-year survival were 0.627, 0.674, 0.677, 0.656, and 0.745, respectively (Figure 4B). For GSE10358, the AUCs indices for 1-,2, 3-, and 4-year survival were 0.669, 0.618, 0.680, and 0.642, respectively (Figure 4C). As well, patients with high-risk score experienced worse clinical outcomes (Figures 4D–F). The relationship between characteristics of AML patients from different datasets and low- and high-risk score were displayed in the Supplementary Table S7. The results confirmed the effectiveness of the 12-gene signature in prognosis prediction. To test the predictive power of the 12-gene expression signature, we employed multiple outstanding models constructed by other researchers for comparison. They are Li’s 24-gene model (Li et al., 2013), Ng’s 17-gene model correlated with leukemia hematopoietic stem cells (LSC) (Ng et al., 2016), Elsayed's 5-gene model (Elsayed et al., 2024), Fu’s 8-gene model (Fu et al., 2021), and Chen’s 4-gene model (Chen et al., 2021). The result noted that the 12-gene signature had the highest C-index for the ROC curves compared to other predictive models in the TCGA AML cohort, indicating the effectiveness of the IMEm7G risk model (Figure 4G). To further identify the role of the gene signature in other cancers, we tested its role using the GEPIA2 online tools (http://gepia2.cancer-pku.cn/) (Tang et al., 2019). The results indicated that the 12-gene signature was predictive of OS in adrenocortical carcinoma (ACC), bladder urothelial carcinoma (BLCA), mesothelioma (MESO), uveal melanoma (UVM), and kidney renal clear cell carcinoma (KIRC) (Supplementary Figure S2A–E). Additionally, the risk model was predictive of disease-free survival (DFS) in ACC, BLCA, lower-grade glioma (LGG), and thyroid carcinoma (THCA) (Supplementary Figure S2F–I). In contrast, for other malignancies profiled in the GEPIA2 database - including head and neck squamous cell carcinoma (HNSC), kidney chromophobe (KICH), breast invasive carcinoma (BRCA), cervical squamous cell carcinoma and endocervical adenocarcinoma (CESC), cholangiocarcinoma (CHOL), colon adenocarcinoma (COAD), diffuse large B-cell lymphoma (DLBC), esophageal carcinoma (ESCA), glioblastoma multiforme (GBM), kidney renal papillary cell carcinoma (KIRP), lower-grade glioma (LGG), liver hepatocellular carcinoma (LIHC), lung adenocarcinoma (LUAD), and additional tumor types - no statistically significant survival differences were observed between high- and low-risk subgroups (Supplementary Table S8).

### The 12-gene signature impacted the immunological features in AML

**FIGURE 5:**
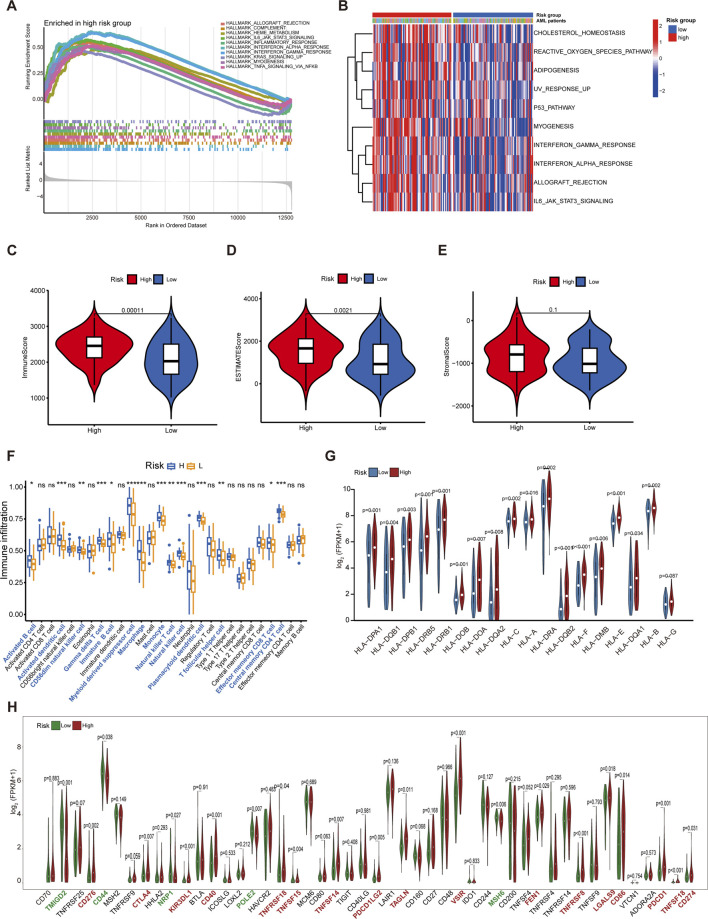
Functional analysis and immune profiles between the low- and high-risk groups. **(A)** GSEA for the high-risk subgroup. **(B)** Heatmaps of the top 10 remarkably different pathways between low- and high-risk groups by GSVA analysis. **(C)** The discrepancies in immune score between high- and low-risk groups. **(D)** The discrepancies in estimate score between high- and low-risk groups. **(E)** The discrepancies in stromal score between high- and low-risk groups. **(F)** The boxplot for immune infiltrating cells among AML patients from different risk groups. **(G)** Comparison of the expression of HLA families in different riskgroups. **(H)** Comparison of the expression of immune checkpoints relevant genes in different risk groups. *p < 0.05; **p < 0.01; ***p < 0.001.

The GSEA and GSVA enrichment analyses were carried out utilizing the Hallmark set to clarify the fundamental mechanisms underlying relevant biological processes. The results demonstrated that immune-related processes, including IL6_Jak_stat3_signaling, interferon gamma response, interferon alpha response, inflammatory response, TNF-α signaling via NFkB, and KRAS signaling were significantly upregulated in the high-risk group compared to the low-risk group (Figures 5A,B). These function enrichment analyses collectively indicated that the high-risk score was strongly associated with immune disorders, which may be a key factor affecting the prognosis of AML patients.

The immune score and estimate score of samples in the high-risk group were significantly higher than those in the low-risk group; in contrast, no significant differences in stromal score were identified between the two groups (Figures 5C–E). To profile the immune infiltrating cells in AML, we conducted the ssGSEA algorithm in the 151 AML samples. Our analysis demonstrated elevated infiltration of 14 out of 28 immune cell subtypes in high-risk patients, most notably myeloid-derived suppressor cells (MDSCs), a key immunosuppressive population that drives tumor progression through promoting immune evasion mechanisms (Tesi, 2019) (Figure 5F). Comparative correlation analysis revealed that MDSCs exhibited significantly stronger associations with immune score than antitumor immune cell populations, underscoring their pivotal role in shaping the immunosuppressive microenvironment of AML patients (Supplementary Figure S5A). We further demonstrated that 18 HLA family genes were significantly differentially expressed between the high-risk and low-risk groups, with most factors being upregulated in the high-risk group (Figure 5G). In addition, we evaluated the expression of 48 immune checkpoint relevant genes. We found that 17 immunomodulators were obviously expressed in the high-risk group compared to the low-risk group, including CD274 (encoding PD-L1), PDCD1 (encoding PD-1), and CTLA4 (Figure 5H).

Consistent positive correlative relationships were identified between m7G regulators and MDSCs infiltration (Supplementary Figure S5B). Risk score and m7G regulators correlation profiling further identified 13 genes with significant associations, and most of which showed distinct expression in the high- and low-risk group (Supplementary Figure S5C,D). Moreover, Notably, NUDT16, LARP1, EIF4E3, and DCPS emerged as significant prognostic indicators for AML survival (Supplementary Figure S5E–H).

### Nomogram deduction by integrating gene expression with clinical indicators

**FIGURE 6:**
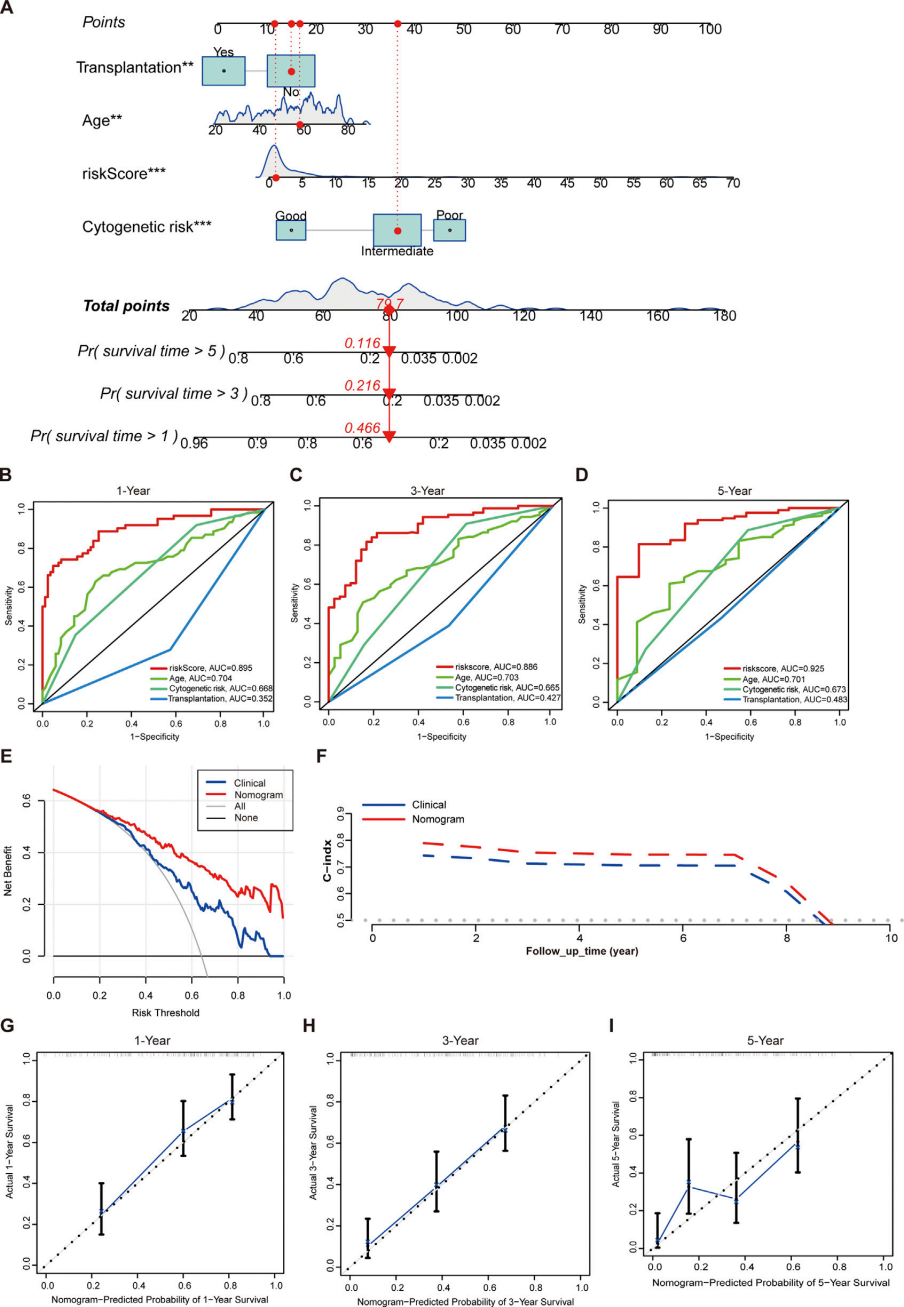
Establishment of a nomogram. **(A)** The nomogram with prognosis forecasting constructed by the gene signature and clinical characters. **(B)** ROC curves of risk score, age, cytogenetic risk, and transplantation treatment for predicting 1-year OS in TCGA cohort. **(C)** ROC curves of risk score, age, cytogenetic risk, and transplantation treatment for predicting 3-year OS in TCGA cohort. **(D)** ROC curves of risk score,age, cytogenetic risk, and transplantation treatment for predicting 5-year OS in TCGA cohort. **(E)** Evaluations of the prediction performance by DCA. “Clinical” means the model constructed by age, cytogenetic risk, and transplantation. **(F)** Evaluations of the prediction performance by time-dependent C-index. “Clinical” means the model constructed by age, cytogenetic risk, and transplantation. **(G)** Evaluations of the 1-year OS prediction performance by calibration curves. **(H)** Evaluations of the 3-year OS prediction performance by calibration curves. **(I)** Evaluations of the 5-year OS prediction performance by calibration curves. **p < 0.01; ***p < 0.001.

We further developed a nomogram to predict the OS of AML patients at 1, 3, and 5 years. This nomogram incorporated risk score along with several clinical variables, including age, cytogenetic risk as defined under the National Comprehensive Cancer Network (NCCN) 2011 guidelines (O'Donnell et al., 2012), and transplantation treatment (Figure 6A). In the univariate and multivariate Cox regression analyses, the 12-gene signature revealed a substantial prognostic value for AML (Supplementary Tables S9, S10). We also calculated additional AUC values for the risk score and individual clinical parameters. Our findings indicated that the risk score had the highest AUC compared to the other clinical factors (Figures 6B–D). The DCA showed obvious net benefits, confirming that the prediction model had better accuracy than the one constructed solely from clinical characteristics, including age, cytogenetic risk, and transplantation (Figure 6E). Additionally, we found that the C-index values for the nomogram were higher than those for models based solely on clinical characteristics, including age, cytogenetics, and transplantation (Figure 6F). Furthermore, the calibration plot demonstrated an excellent alignment with the ideal model (Figures 6G–I).

### Analysis of individual mRNA expression for the 12 prognostic genes and identification of effective therapeutics in high-risk AML patients

**FIGURE 7:**
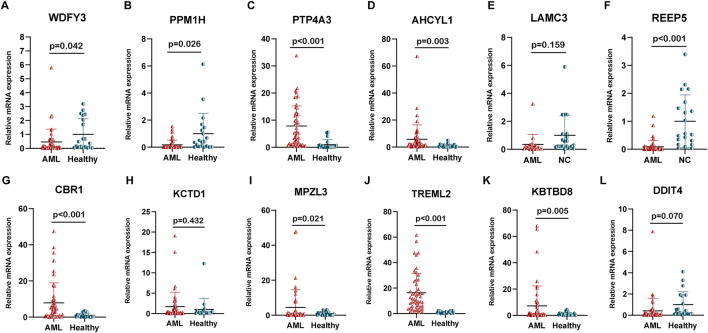
The relative mRNA expression of the 12 prognostic genes. The expression of **(A)** WDFY3, **(B)** PPM1H, **(C)** PTP4A3, **(D)** AHCYL1, **(E)** LAMC3, **(F)** REEP5, **(G)** CBR1, **(H)** KCTD1, **(I)** MPZL3, **(J)** TREML2, **(K)** KBTKD8, and **(L)** DDIT4 in AML patients (n = 50) and healthy donors (n = 20).

**FIGURE 8:**
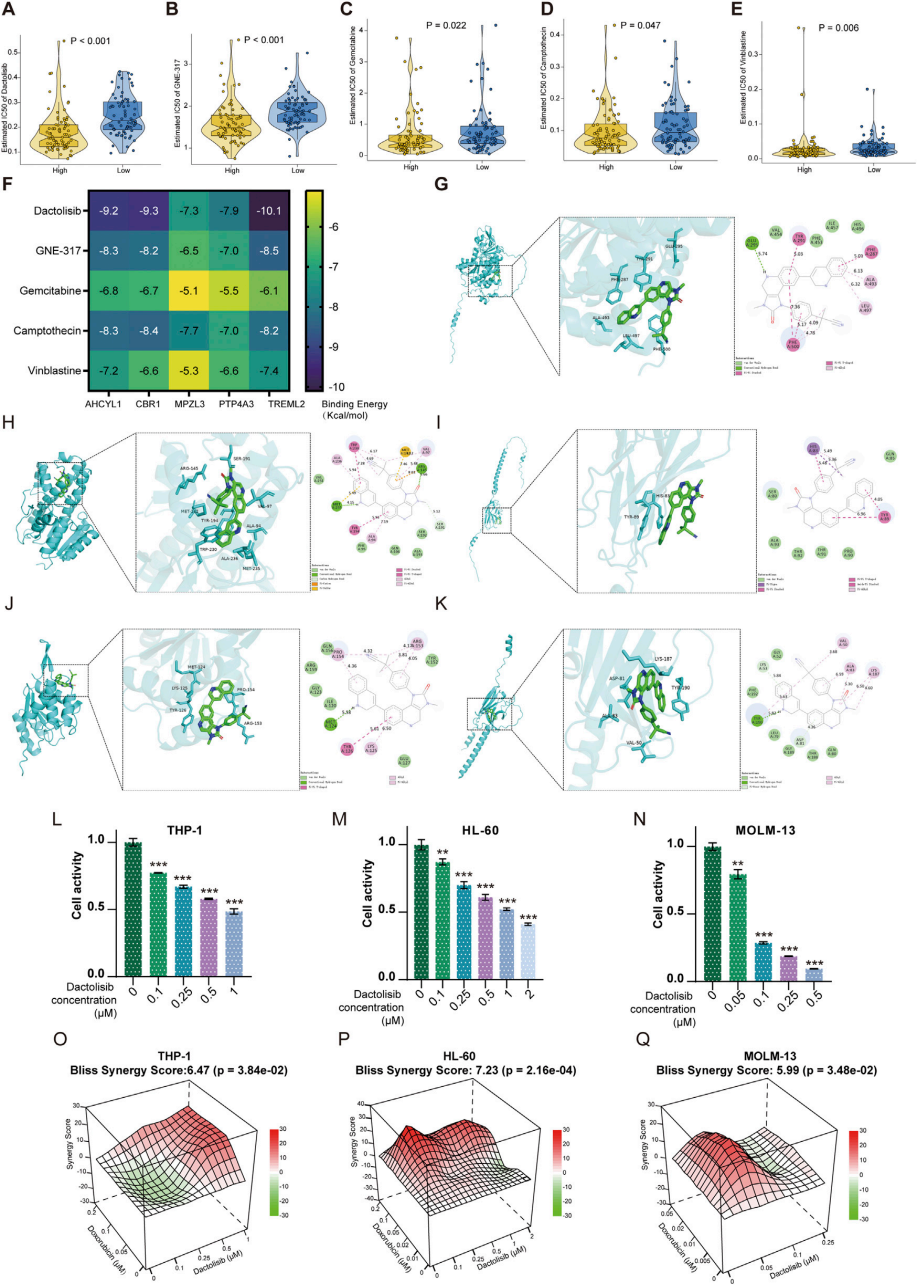
Drug analysis for targeted therapy. **(A)** Dactolisib, **(B)** GNE-317, **(C)** Gemcitabine, **(D)** Camptothecin, and **(E)** Vinblastine were the top 5 predicted sensitive drugs in the high-risk group. **(F)** The binding energy of critical compounds and targets (kcal/mol). The molecular docking between Dactolisib and **(G)** AHCYL1, **(H)** CBR1, **(I)** MPZL3, **(J)** PTP4A3, and **(K)** TREML2 encoded proteins. The increased concentration of Dactolisib decreased the viability of AML cells, including **(L)** THP-1, **(M)** HL-60, and **(N)** MOLM-13. Synergism was calculated for the drug combinations of Dactolisib with Doxorubicin in **(O)** THP-1, **(P)** HL-60, and **(Q)** MOLM-13 cells. The synergy score was computed using the Bliss Independence Model and depicted as a heatmap. Positive Bliss Scores are indicative of synergism. **p < 0.01; ***p < 0.001.

We carried out qPCR to validate our bioinformatic results. The result of qPCR illustrated that in comparison with healthy donors, the expression of WDFY3, PPM1H, and REEP5 was significantly lower, while that of PTP4A3, AHCYL1, CBR1, MPZL3, TREML2, and KBTBD8 was higher in AML patients (Figures 7A–L). The “oncoPredict” R project was utilized to predict the half maximal inhibitory concentration (IC50) of each drug for AML patients between high and low-risk groups using the GDSC2 database (https://www.cancerrxgene.org/). As illustrated in Figures 8A–E, the five drugs with the highest sensitivity for high-risk patients, compared to the low-risk group, were dactolisib, GNE-317, gemcitabine, camptothecin, and vinblastine. Dactolisib and GNE-317 are PI3K/mTOR inhibitors known for their promising clinical utility. Notably, dactolisib exhibited vital antitumor activity and the potential to augment immune response (Hu et al., 2021; Tehranian et al., 2022). The other three drugs, gemcitabine, camptothecin, and vinblastine, are chemotoxic agents used in chemotherapy for various tumors (Wander et al., 2020; Li et al., 2021b; Wang et al., 2023b). We further conducted molecular docking analyses of these drugs with proteins associated with unfavorable prognoses, including PTP4A3, AHCYL1, TREML2, CBR1, and MPZL3 using the AutoDock software. The findings demonstrated that all of these pharmaceuticals displayed a binding affinity for the specified therapeutic targets (Figure 8F), implying that the mechanism through which these drugs treat AML was probably by the interactions with these critical targets. Due to the heterogeneity of AML, the effectiveness of conventional chemotherapy regimens has been limited. As a result, targeted therapies are increasingly being utilized in both newly diagnosed and relapsed/refractory (R/R) AML cases (Kantarjian et al., 2021). Dactolisib was the targeted drug with the strongest interaction among these molecules, characterized by the lowest binding energy. Additionally, it demonstrated tumor-killing effects in various solid tumors, while its role in AML remains to be explored. We further explored the role of dactolisib in AML. As shown in Figures 8G–K, all therapeutic agents exhibited interactions with dactolisib at specific binding regions. We then treated AML cells with dactolisib as a monotherapy and detected the viability of AML cells. As the concentration of dactolisib increased, the viability of AML cells decreased (Figures 8L–N). Doxorubicin has been a first-line treatment for newly diagnosed AML patients for decades; however, its effectiveness is often limited due to the development of resistance. Combining doxorubicin with targeted agents might improve the chemosensitivity of doxorubicin in the clinic (Leung et al., 2020; Gui et al., 2021). To investigate whether dactolisib and doxorubicin have a synergistic effect, we treated AML cells with both drugs in combination. Using the SynergyFinder tool, we found that dactolisib and doxorubicin exhibited significant synergy (Figures 8O–Q), suggesting a novel therapeutic option for AML treatment. Further studies are needed to elucidate the underlying mechanism of this combination effect.

## Discussion

Initially, we attempted to build the predictive model using either m7G-related or IME-related genes separately. However, regarding prediction performance, either the IME-related model or the m7G-related model exhibited obvious demerits. The role of combining m7G methylation and IME for risk stratification and therapy guidance is still obscure in AML. On that account, our study investigated IME and m7G-related DEGs, aiming to uncover their joint roles and contribute to new prognostic models and treatment strategies for AML patients. By examining the distinct categories and subclasses of AML patients via IME- and m7G-associated DEGs, we discovered that individuals in the m7G-related cluster2 and cluster3 had comparable survival rates throughout 1–3 years when compared to those in the IME-related cluster 2 and cluster3. Not only that, there were significant differences in immune score among the distinct m7G-relevant clusters. Patients in cluster3 and cluster2 showed higher immune score than those in cluster1. These findings suggested a correlation between m7G and IME, indicating that distinct classifications based on m7G revealed variations in IME and AML survival. Additionally, they imply that m7G may interact with IME to influence treatment strategies. Currently, the IME and m7G-related model is rarely used in tumor prognosis and has not been reported in the context of AML.

Our model performed excellently in predicting the prognosis of AML. Based on their risk score, we were able to distinguish AML patients into two risk groups, with the low-risk group exhibiting better survival rates. The risk score demonstrated a strong ability to independently predict OS. In the high-risk group, there were a greater proportion of elderly patients, as well as a higher percentage of individuals with intermediate or poor cytogenetic stratification and those who had not undergone transplantation. The prognostic scoring model comprised 12 characteristic genes, including MPZL3, TREML2, PTP4A3, AHCYL1, CBR1, REEP5, PPM1H, WDFY3, LAMC3, KCTD1, DDIT4, and KBTBD8. The 12 genes were taken as a whole in this study to determine risk stratification, IME characteristics, and responsiveness to anticancer pharmacological agents within the AML cohort. Among these genes, the MPZL3 gene encodes a membrane protein that is crucial for several biological processes, including epidermal differentiation (Bhaduri et al., 2015), mitochondrial dysfunction (Wikramanayake et al., 2022), and immune infiltrating (Guo et al., 2024). Abnormal expression of MPZL3 is involved in the development of lung carcinoma (Stern et al., 2022). However, the role of MPZL3 in AML is limited. TREML2 is known to influence inflammatory responses by modulating the microglia polarization and the NLRP3 inflammasome (Wang et al., 2023a). Current studies suggest that overexpression of PTP4A3 may stimulate the AKT and WNT/β-catenin signaling pathways, enhancing the viability of AML cells (Zhou et al., 2018). AHCYL1 plays a dual role in tumors; it acts as a tumor suppressor in gastric, ovarian, and breast cancers while promoting chemotherapy resistance in malignant melanoma (Nakazawa et al., 2019; Jeong et al., 2012; Chen et al., 2024; Wittig et al., 2002). The increased expression of CBR1 in adipocytes may metabolize daunorubicin, thereby reducing the effectiveness of the therapy (Sheng et al., 2017). By regulating reactive oxygen species production, elevated expression of CBR1 is able to safeguard leukemia cells against As2O3. Conversely, the loss of CBR1 significantly increases the cells’ susceptibility to As2O3(Jang et al., 2012). REEP5 has been recognized as a binding partner of CXCR1, subsequently triggering the IL-8-CXCR1/2 signaling pathway and facilitating the growth and metastasis of diverse tumor cell types (Fan et al., 2022). PPM1H is regarded as a tumor suppressor across various malignancies. Loss of PPM1H confers trastuzumab resistance by reducing the protein levels of the tumor suppressor p27 in breast cancer (Lee-Hoeflich et al., 2011). Low expression level of PPM1H is associated with worse outcomes in hepatocellular carcinoma, colorectal cancer, and pancreatic cancer (Iyer et al., 2023; Xu et al., 2019; Zhu et al., 2016). WDFY3 interacts with PML-RARA, promoting the autophagy-mediated degradation of PML-RARA (Du et al., 2021). WDFY3 also engages with GABARAP to facilitate the degradation of apoptotic cellular components, thereby supporting the maintenance of tissue homeostasis (Shi et al., 2022). The roles of LAMC3 and KCTD1 are essential in the development and differentiation of the nervous system (Zhu et al., 2024; Liao and Muntean, 2024), while recent studies on its involvement in carcinoma have yet to be sufficient. Overexpression of DDIT4 induced by serum starvation inhibits the transcription activity of mTORC1, thereby abrogating the oncogenic potential of melanoma cells (Hwang et al., 2024). DDIT4 has also been discovered as a downstream target of phenformin, which promotes autophagic and apoptotic cell death to suppress the growth of oral squamous cell carcinoma (Zhuang et al., 2024). KBTBD8 is served as a critical regulator of cell-fate determination (Werner et al., 2015). In summary, existing research indicates that most of the genes identified in this model have yet to be elucidated regarding their functions and mechanisms in AML. While many of these genes show a significant impact on OS or EFS in the TCGA AML cohort, further investigations are needed to clarify the roles and mechanisms of genes such as MPZL3, TREML2, and AHCYL1 in the context of AML. This will provide a stronger theoretical foundation for prognostic evaluation and assist in identifying novel therapeutic targets for AML treatment.

Genetic mutations play a crucial role in leukemogenesis and the development of drug resistance. We characterized the tumor mutation landscape in high-risk and low-risk groups, revealing a significant distinction between these categories. The mutation frequency in the high-risk group was notably higher than in the low-risk group, particularly for mutations in genes such as DNMT3A, NPM1, and TP53. Previous studies have shown that clonal mutations in DNMT3A, NPM1, and TP53 are linked to treatment resistance. Additionally, research indicates that DNMT3A mutations contribute to anthracycline resistance by impairing nucleosome remodeling in AML (Guryanova et al., 2016). NPM1 mutations are prevalent in AML, and patients with NPM1 mutations respond well to conventional chemotherapy (Falini et al., 2021). However, around 50% of these patients eventually experience disease progression, particularly those with additional mutations (Ranieri et al., 2022). DNMT3A or FLT3 mutations alongside NPM1 are associated with inferior survival outcomes (Yao et al., 2024). TP53 mutations are frequently linked to unfavorable outcomes and diminished therapeutic response (Kadia et al., 2016). Vadakekolathu et al. pointed out that TP53 mutation was correlated with enhanced immune infiltration, high levels of PD-L1, and the upregulated intermediates involved in PI3K-Akt signaling, NF-κB pathway, JAK/STAT signaling, and IFN-γ pathway (Vadakekolathu et al., 2020). These further strengthens the prognostic credibility of the risk score assessment.

Through comprehensive GSEA and GSVA analyses of enriched pathways and biological functions, we discovered that most of the differences were primarily related to immune responses. Notable pathways activated in the high-risk group included IL6/JAK/STAT3 signaling, interferon gamma and alpha responses, TNF-α signaling via NF-kB, and KRAS signaling. Previous research has shown that these pathways exhibit aberrant hyperactivation across various cancer types. The abnormal activation of these pathways may contribute to tumorigenesis, progression, and therapy resistance by altering the immune microenvironment (Johnson et al., 2018; Litak et al., 2019; De Simone et al., 2015; Hu et al., 2023). This finding aligns with our observations that patients in the high-risk group have relatively poor clinical outcomes compared to those in the low-risk group. However, the interplay between these aberrantly activated processes and m7G modification has rarely been explored to date.

Notably, our analyses revealed that high-risk patients exhibited significantly enriched MDSCs and monocyte infiltration compared to low-risk counterparts. Concurrently, this cohort demonstrated upregulation of key immune checkpoint molecules, including PD-1, PD-L1, and CTLA-4, which are closely associated with immune suppression (Bronte et al., 2016; Zhang et al., 2021). Paradoxically, despite elevated natural killer (NK) cell levels were critical mediators of immune surveillance (Huntington et al., 2020), these cells exhibited functional impairment via MDSC-mediated crosstalk (Joshi and Sharabi, 2022; Neo et al., 2024). Human leukocyte antigen (HLA) dysregulation further contributed to immune evasion in high-risk patients. Liu et al. indicated that overexpression of HLA-E in circulating tumor cells (CTC) engaged CD94-NKG2A inhibitory receptors on NK cells, blunting antitumor responses (Liu et al., 2023). The HLA-G expression is upregulated in various tumor cells and is rarely observed in healthy tissues (Loustau et al., 2020). The tolerogenic HLA-G molecule mediates tumor immune evasion by binding to inhibitory receptors on the surface of immune effector cells (Wang et al., 2024). These findings indicated that the high-risk cohort was characterized by immune suppressive factors facilitating immune evasion. Integrative analysis identified strong correlations between MDSC abundance and m7G regulators expression. We posited that these m7G-associated effectors may drive AML progression through coordinated regulation of MDSCs recruitment/activation and immunosuppressive niche formation, ultimately governing disease progression and therapeutic resistance. This suggested that immunotherapeutic agents targeting these factors may be effective for individuals with elevated risk score.

In addition, we established a nomogram integrating risk score with clinical features to explore clinical application values further. According to the analyses of ROC, DCA, calibration curves, and C-index, the nomogram that includes the risk score still has unique advantages that are far superior to the conventional clinical factors.

Our model not only focused on risk stratification and immunotherapy studies but also provided insights into sensitivity to certain anticancer drugs. The most sensitive predictive drugs for high-risk groups are dactolisib, GNE-317, camptothecin, gemcitabine, and vinblastine. Among these pharmaceuticals, dactolisib functions as a dual ATP-competitive inhibitor targeting PI3K and mTOR signaling pathways, exhibiting substantial antitumor efficacy and remarkable synergistic effects with other agents in preclinical investigations (Deng et al., 2023; Çetin et al., 2023; Li et al., 2021a; Liu et al., 2022). Not only that, the administration of dactolisib was able to remodel the tumor microenvironment (TME) by enhancing the prevalence of anti-tumor immune cells, including T-CD4 cells and M1 macrophages, while simultaneously reducing the population of immune suppressive cells, including MDSCs and also M2 macrophages (Taghiloo et al., 2023; Li et al., 2020). Our *in vitro* study indicated that the monotherapy of dactolisib effectively eradicated leukemia cells. Furthermore, combing dactolisib with doxorubicin exhibited synergistic efficacy in AML cells.

## Conclusion

In summary, our study integrated IME and m7G to develop a 12-gene prognostic model demonstrating robust predictive capability. Moreover, the potential targeted medication, dactolisib, was predicted for the high-risk group and revealed antileukemic activity as monotherapy and synergetic effect combined with doxorubicin, an agent included in the first-line “3 + 7” strategy.

## Data Availability

The datasets generated and/or analyzed during the current study are available in the TCGA database (https://portal.gdc.cancer.gov/), Beat-AML database (http://www.vizome.org/), and the GEO database (https://www.ncbi.nlm.nih.gov/gds/), accession number GSE10358 (https://www.ncbi.nlm.nih.gov/geo/query/acc.cgi?acc=GSE10358) and GSE71014 (https://www.ncbi.nlm.nih.gov/geo/query/acc.cgi?acc=GSE71014).

## Supplementary material

SUPPLEMENTARY FIGURE S1
**(A)** The GO analysis of IME-relevant DEGs. **(B)** The KEGG analysis of IME-relevant DEGs. **(C)** ROC curves of IME-relevant model for predicting 1-, 2-, 3-, 4-, and 5-year OS in TCGA AML cohort. **(D)** ROC curves of IME-relevant model for predicting 1-, 2-, 3-, 4-, and 5-year OS in the Beat-AML cohort. **(E)** ROC curves of IME-relevant model for predicting 1-, 2-, 3-, 4-, and 5-year OS in the GSE71014 cohort. **(F)** ROC curves of IME-relevant model for predicting 1-, 2-, 3-, and 4-year OS in the GSE10358 cohort. **(G)** ROC curves of m7G-relevant model for predicting 1-, 2-, 3-, 4-, and 5-year OS in TCGA cohort. **(H)** ROC curves of m7G-relevant model for predicting 1-, 2-, 3-, 4-, and 5-year OS in the Beat-AML cohort. **(I)** ROC curves of m7G-relevant model for predicting 1-, 2-, 3-, 4-, and 5-year OS in the GSE71014 cohort. **(J)** ROC curves of m7G-relevant model for predicting 1-, 2-, 3-, and 4-year OS in the GSE10358 cohort. **(K)** The cophenetic correlation coefficient, rss, and silhouette were for optimal number of IME based subtypes. **(L)** The cophenetic correlation coefficient, rss, and silhouette were for optimal number of m7G based subtypes. **(M)** The distribution of immune score in the m7G-based distinct clusters. **(N)** Clustering based on expression data of TCGA-LAML, with 10000 as the threshold, two outliers were removed.

SUPPLEMENTARY FIGURE S2 KM survival curves of the model predicting the prognostic rate of OS in **(A)** adrenocortical carcinoma (ACC), **(B)** bladder urothelial carcinoma (BLCA), **(C)** mesothelioma (MESO), **(D)** uveal melanoma (UVM), and **(E)** kidney renal clear cell carcinoma (KIRC). KM survival curves of the model predicting the prognostic rate of DFS in **(F)** adrenocortical carcinoma (ACC), **(G)** bladder urothelial carcinoma (BLCA), **(H)** lower-grade glioma (LGG), and **(I)** thyroid carcinoma (THCA).

SUPPLEMENTARY FIGURE S3
**(A)** The correlation between MDSCs infiltration and immune score, **(B)** Association of 29 m7G regulatory gene expression with MDSC infiltration, **(C)** The correlation between risk score and the expression of 29 m7G relevant genes, **(D)** The expression of 29 m7G regulators in the high- and low-risk groups, **(E)** The survival analysis of NUDT16 in AML patients, **(F)** The survival analysis of LARP1 in AML patients, **(G)** The survival analysis of EIF4E3 in AML patients, **(H)** The survival analysis of DCPS in AML patients.

SUPPLEMENTARY TABLE S1 The primers designed for qPCR analysis.

SUPPLEMENTARY TABLE S2 The clinical characteristics of patients in the FF and WW groups.

SUPPLEMENTARY TABLE S3 Survival related DEGs screened from WGCNA analysis.

SUPPLEMENTARY TABLE S4 The detailed genes screened from univariate Cox regression analysis.

SUPPLEMENTARY TABLE S5 The VIF values of the 12 prognostic genes.

SUPPLEMENTARY TABLE S6 The PH hypothesis for the 12 prognostic genes.

SUPPLEMENTARY TABLE S7 The relationship between characteristics of AML patients from different datasets and risk score.

SUPPLEMENTARY TABLE S8 Pan-Cancer Prognostic Utility of the 12-Gene Signature.

SUPPLEMENTARY TABLE S9 The performance of univariate Cox regression analysis with risk score and clinical variables, including age, FAB classification, Race, gender, cytogenetic risk stratification, and transplantation treatment.

SUPPLEMENTARY TABLE S10 The performance of multivariate Cox regression analysis with risk score and clinical variables, including age, cytogenetic risk stratification, and transplantation treatment.

## Glossary

AML: Acute myeloid leukemia
OS: Overall survival
EFS: Event-free survival
DFS: disease-free survival
TME: Tumor microenvironment
TIME: Tumor immune microenvironment
IME: Immune microenvironment
TCGA: The Cancer Genome Atlas
GEO: Gene Expression Omnibus
DEGs: Differentially expressed genes
GSEA: Gene set enrichment analysis
HSCT: Hematopoietic stem cell transplantation
OD: optical density
ESTIMATE: The Estimation of Stromal and Immune cells in Malignant Tumour tissues using Expression data
NMF: Non-negative matrix factorization
WGCNA: Weighted correlation network analysis
GO: Gene ontology
KEGG: Kyoto Encyclopedia of Genes and Genomes
FDR: False discovery rate
KM: Kaplan-Meier
LASSO regression: Least absolute shrinkage and selection operator regression
ROC: Receiver operating characteristic
AUCs: Area under the curve
DCA: Decision curve analysis
C-index: Concordance index
qPCR: quantitative real-time PCR
IC50: Half maximal inhibitory concentration
NES: Normalized enrichment score
HR: Hazard ratio
NCCN: National Comprehensive Cancer Network
FC: Fold change
TOM: Topological overlap matrix
PDCD1: Programmed cell death 1
CD274: cluster of differentiation 274
CTLA4: Cytotoxic T-lymphocyte associated protein 4
MDSC: myeloid-derived suppressor cells
NK cells: Natural killer cells
CTC: Circulating tumor cells
ACC: Adrenocortical carcinoma
BLCA: Bladder urothelial carcinoma
MESO: Mesothelioma
UVM: Uveal melanoma
KIRC: Kidney renal clear cell carcinoma
LGG: Lower-grade glioma
THCA: Thyroid carcinoma
HNSC: Head and neck squamous cell carcinoma
KICH: Kidney chromophobe
BRCA: Breast invasive carcinoma
CESC: Cervical squamous cell carcinoma and endocervical adenocarcinoma
CHOL: Cholangiocarcinoma
COAD: Colon adenocarcinoma
DLBC: Diffuse large B-cell lymphoma
ESCA: Esophageal carcinoma
GBM: Glioblastoma multiforme
KIRP: Kidney renal papillary cell carcinoma
LIHC: Liver hepatocellular carcinoma
LUAD: Lung adenocarcinoma
LAMC3: Laminin subunit gamma 3
KCTD1: Potassium channel tetramerization domain containing 1
PPM1H: Protein phosphatase, Mg2+/Mn2+ dependent 1H
WDFY3: WD repeat and FYVE domain containing 3
REEP5: Receptor accessory protein 5
DDIT4: DNA damage inducible transcript 4
PTP4A3: Protein tyrosine phosphatase 4A3
AHCYL1: Adenosylhomocysteinase like 1
TREML2: Triggering receptor expressed on myeloid cells like 2
KBTBD8: Kelch repeat and BTB domain containing 8
CBR1: carbonyl reductase 1
MPZL3: Myelin protein zero like 3
