# Supplementary figures and images for: The joint role of the immune microenvironment and N7-methylguanosine for prognosis prediction and targeted therapy in acute myeloid leukemia
Source: Front Genet. 2025 Jun 13;16:1540992. doi: 10.3389/fgene.2025.1540992 (PMC12202357; doi:10.3389/fgene.2025.1540992)

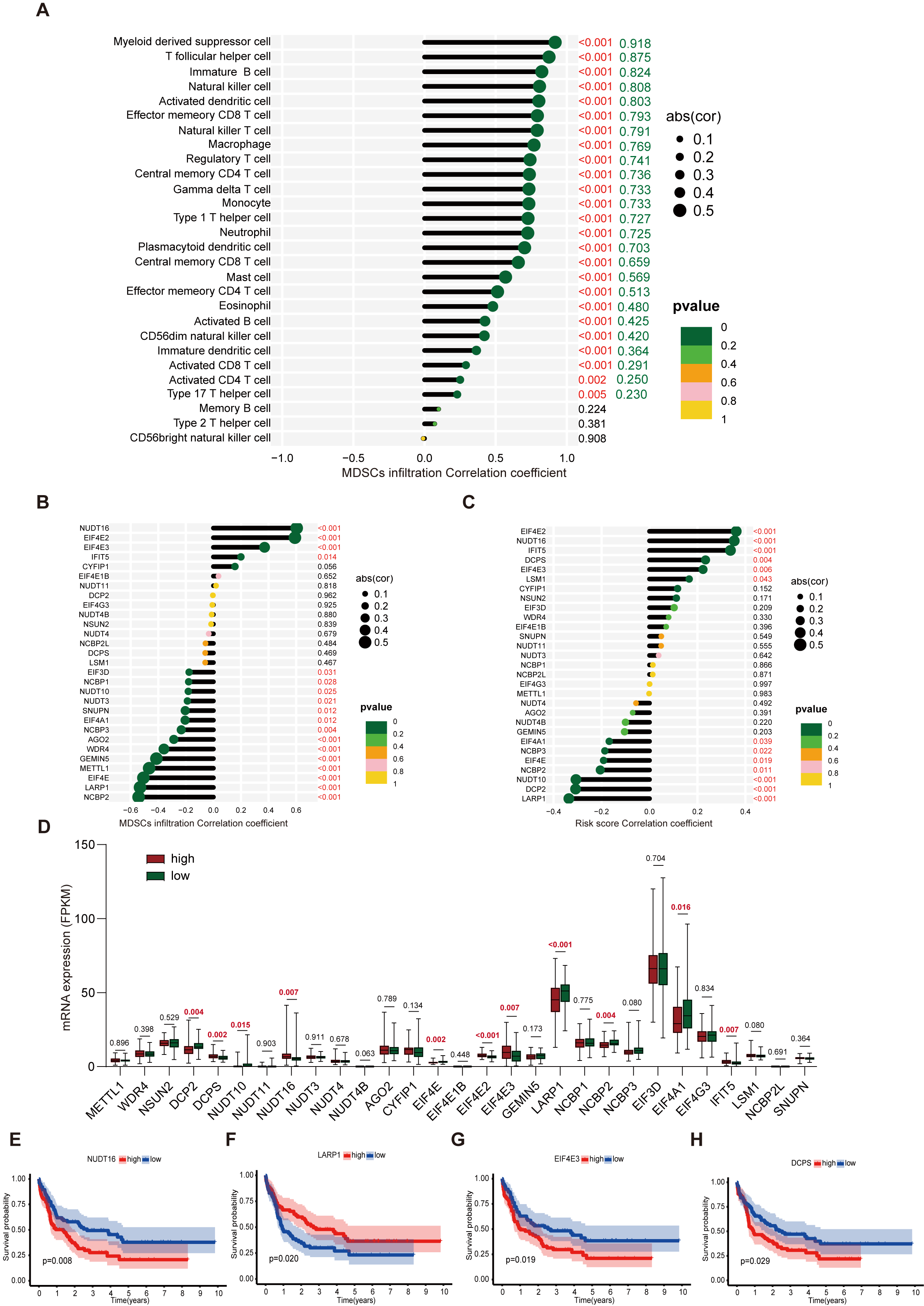

Supplement: Supplementary file 1 [file Image3.tif]

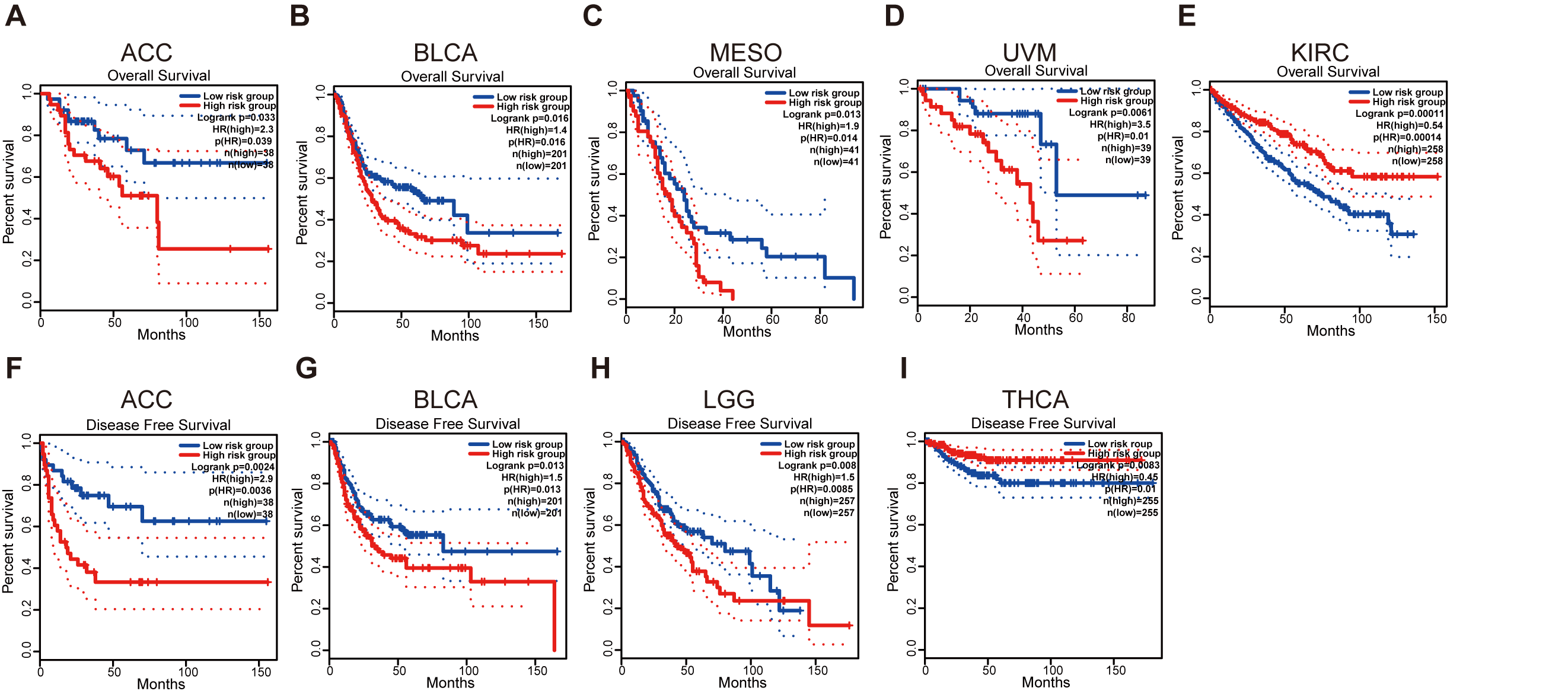

Supplement: Supplementary file 2 [file Image2.tif]

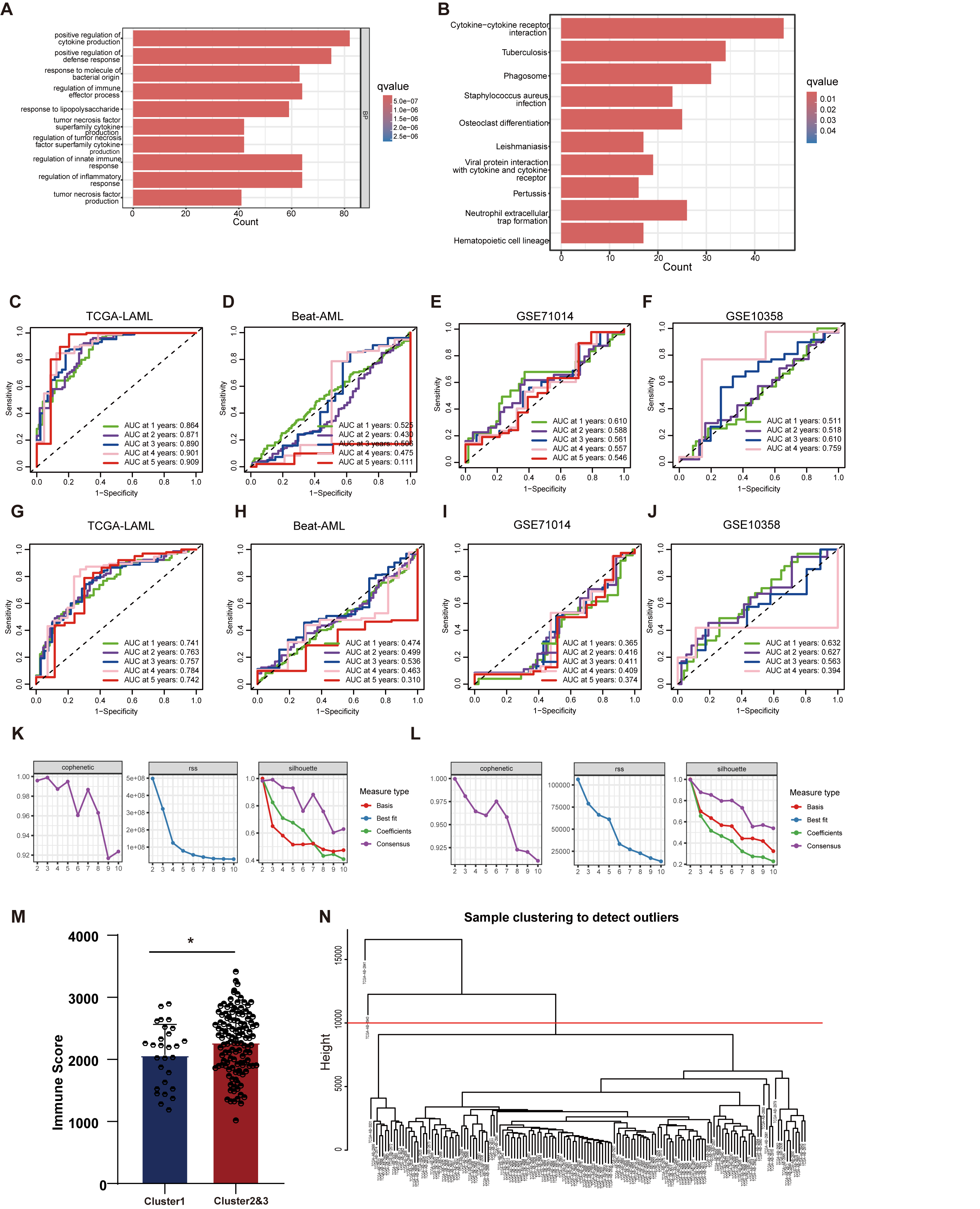

Supplement: Supplementary file 3 [file Image1.tif]
